# FLRT2 suppresses bladder cancer progression through inducing ferroptosis

**DOI:** 10.1111/jcmm.17855

**Published:** 2023-07-21

**Authors:** Pengcheng Jiang, Jinzhuo Ning, Weimin Yu, Ting Rao, Yuan Ruan, Fan Cheng

**Affiliations:** ^1^ Department of Urology Renmin Hospital of Wuhan University Wuhan China

**Keywords:** ACSL4, bladder cancer, ferroptosis, FLRT2

## Abstract

Bladder cancer is a common tumour worldwide and exhibits a poor prognosis. Fibronectin leucine rich transmembrane protein 2 (FLRT2) is associated with the regulation of multiple tumours; however, its function in human bladder cancer remain unclear. Herein, we found that FLRT2 level was reduced in human bladder cancer and that higher FLRT2 level predicted lower survival rate. FLRT2 overexpression inhibited, while FLRT2 silence facilitated tumour cell growth, migration and invasion. Mechanistic studies revealed that FLRT2 elevated acyl‐CoA synthetase long‐chain family member 4 (ACSL4) expression, increased lipid peroxidation and subsequently facilitated ferroptosis of human bladder cancer cells. In summary, we demonstrate that FLRT2 elevates ACSL4 expression to facilitate lipid peroxidation and subsequently triggers ferroptosis, thereby inhibiting the malignant phenotype of human bladder cancer cells. Overall, we identify FLRT2 as a tumour suppressor gene.

## 1 INTRODUCTION

Bladder cancer is a common tumour worldwide, and accounts for an approximately 573 thousands new patients and 212 thousands mortality in 2020. In addition, 25% of the patients are diagnosed as muscle‐invasive bladder cancer that exhibits higher metastasis and mortality risks.^1^, ^2^, ^3^ Multiple mechanisms are implicated in the pathogenesis of bladder cancer, among which, ferroptosis plays an indispensable role in bladder cancer progression. Ferroptosis is an iron‐dependent form of lipid peroxidation‐mediated cell death. The imbalance of intracellular reactive oxygen species (ROS) production and degradation leads to the decrease of cell antioxidant capacity, lipid peroxidation overload and plasma membrane rupture and finally leads to cell death.^4^, ^5^, ^6^ Sun et al.^7^ revealed that inducing ferroptosis could effectively kill bladder cancer cells. In contrast, inhibiting ferroptosis facilitated the survival, migration and invasion of human bladder cancer cells.^8^ Therefore, dissecting the mechanism of ferroptosis in the tumorigenesis of bladder cancer and subsequently finding its activators are crucial for developing novel therapeutic approaches.

Fibronectin leucine rich transmembrane protein 2 (FLRT2), a member of the FLRT family of proteins, initially represents a novel family of chemorepellents for Unc5‐positive neurons and modulates the migration of cortical neurons.^9^ In addition, FLRT2 is exclusively expressed in the fully delaminated epicardium and determines heart morphogenesis.^10^ Yet, emerging evidences have indicated that FLRT2 also participates in regulating tumour progression. By analysing whole genome messenger RNA profiling on the Cancer Genome Atlas, Dai et al.^11^ found a correlation between FLRT2 level and the survival rate of gastric cancer patients. Wu et al.^12^ found that differentially methylated FLRT2 correlated with the Gleason score in prostate cancers. Bae et al.^13^ demonstrated that FLRT2 hypermethylation was associated with FLRT2 downregulation, thereby increasing the proliferation and migration of breast cancer cells. Similarly, Guo et al.^14^ showed that FLRT2 was downregulated in colorectal cancer tissues and cells by DNA methylation, and that FLRT2 overexpression significantly inhibited the migration and invasion of human colorectal cancer cells. Herein, we intend to explore the function of FLRT2 in bladder cancer.

## 2 MATERIALS AND METHODS

### 2.1 Chemicals

Ferrostain‐1 (Fer‐1, #S7243, an inhibitor of ferroptosis), liproxstatin‐1 (Lip‐1, #S7699, an inhibitor of ferroptosis), necrostatin‐1 (Nec‐1, #S8087, an inhibitor of necroptosis), Z‐VAD‐FMK (Z‐VAD, #S7023, an inhibitor of apoptosis), chloroquine (CQ, #S6999; an inhibitor of autophagy) and 2′, 7’‐Dichlorodihydrofluorescein diacetate (DCFH‐DA, #S9687) were purchased from Selleck (Houston, TX, USA). Cell Counting Kit 8 (CCK8) (#ab228554), Lactate Dehydrogenase (LDH) Assay Kit (#ab102526), Lipid Peroxidation Assay Kit (#ab118970), 12(S)‐hydroxyeicosatetraenoic acid (HETE) ELISA Kit (#ab133034), 15(S)‐HETE ELISA Kit (#ab133035), Iron Assay Kit (#ab83366) and Glutathione (GSH) Assay Kit (#ab239727) were purchased from Abcam (Cambridge, UK). CytoSelect™ 24‐Well Cell Migration and Invasion Assay Combo Kit (#CBA‐100) was purchased from Cell Biolabs (San Diego, CA, USA).

### 2.2 Cells and treatments

Human bladder cancer cells (5637, T24 and UMUC‐3) and immortal ureteral epithelium cells (SV‐HUC‐1) were obtained from the ATCC (Manassas, VA, USA). Human BC cell lines T24 and 5637 were maintained in RPMI‐1640 medium (Pricella). UMUC‐3 cells were grown in DMEM (Pricella). SV‐HUC‐1 cells were cultured in F‐12 K medium (Pricella). All culture media were supplemented with 10% fetal bovine serum (FBS, Gibco).^15^, ^16^ All cells were cultured in a humidified incubator at 37°C and 5% CO_2_. To overexpress FLRT2, T24 and UMUC‐3 cells were infected with lentivirus carrying FLRT2 (NM_013231, FLRT2 OE; #SC115333, OriGene) at a multiplicity of infection (MOI) of 30 or control (CTRL) plasmids for 6 h, and then cultured for an additional 72 h before harvested. To knock down FLRT2, bladder cancer cells were infected with lentivirus carrying short hairpin RNA against FLRT2 (MOI = 50, shFLRT2; #TL312965V, OriGene) or shCtrl for 6 h. For acyl‐CoA synthetase long‐chain family member 4 (ACSL4) silence, bladder cancer cells were infected with FLRT2 OE for 6 h, cultured in fresh DMEM containing 10% FBS for 24 h, and then transfected with small interfering RNA against ACSL4 (siACSL4, #SR320122, OriGene) at a MOI of 40 for 4 h. Next, cells were cultured in fresh medium for an additional 48 h. In addition, FLRT2‐overexpressed bladder cancer cells were also incubated with Fer‐1 (1 μmol/L), Lip‐1 (0.2 μmol/L), Nec‐1 (10 μmol/L), Z‐VAD (10 μmol/L) or CQ (25 μmol/L) to inhibit ferroptotic, apoptotic, necroptotic or autophagic cell death, respectively as previously described.^17^

### 2.3 Western blot

Bladder cancer cells were washed with ice‐cold phosphate buffered saline (PBS), lysed in RIPA lysis buffer supplemented with protease and phosphatase inhibitors, and the concentration was measured by BCA Protein Assay Kit (Invitrogen).^18^, ^19^ For western blot analysis, 30 μg of proteins were loaded onto 10% SDS‐PAGE and transferred to PVDF membranes. Next, the membranes were blocked with 5% milk for 1 h at room temperature, and incubated with anti‐FLRT2 (#ab154023, Abcam), anti‐ACSL4 (#ab155282, Abcam) or anti‐β‐actin (#ab8226, Abcam) overnight at 4°C. On the next day, the sample was then washed thrice with TBST for 10 min each time. The membrane was removed and incubated with the peroxidaseconjugated secondary antibody (ProteinTech Group) at 37°C for 2 h. Finally, ECL (Biosharp Life Sciences) colour was developed with β‐actin as an internal reference to analyse the protein expression level on the membrane and visualized with the by the chemiluminescence system (ChemiDocTM Touch; Bio‐Rad).

### 2.4 Cell growth and LDH releases analysis

To detect cell growth, transfected cells were plated into the 96‐well plates at a cell density of 2 × 10^3^ cells/well, and then cell viability at the 1st, 2nd and 3rd day was detected using a CCK‐8 kit as previously described.^20^, ^21^, ^22^For the analysis of LDH releases, cell medium and cells at the 3rd day were collected and subjected to LDH detection using a LDH Assay Kit according to the manufacturers' instructions. Briefly, supernatants of cell medium and cell lysates were prepared, and mixed with 50 μL of Reaction Mix containing 48 μL of LDH Assay Buffer and 2 μL of LDH Substrate Mix. Then, the optical density (OD) values were measured at 450 nm.^23^ LDH releases were calculated as: Medium LDH/(Medium LDH + Cell lysates LDH).

### 2.5 Cell migration and invasion assay

Cell migration and invasion were measured using a colorimetric CytoSelect™ 24‐Well Cell Migration and Invasion Assay Combo Kit according to the manufacturers' instructions. Briefly, transfected cells in serum‐free medium were plated into the inside of each insert of the 24‐well plates at a cell density of 2 × 10^5^ cells/insert, and 500 μL of the medium containing 10% FBS was added to the lower well of the migration plate as a chemoattractant. After 12 h, the non‐migratory cells were gently removed by the cotton‐tipped swabs, and the insert was transferred to 400 μL of Cell Stain Solution and allowed for an incubation of 10 min at room temperature. Next, the inserts were dried and transferred to 200 μL of Extraction Solution and allowed for an incubation of 10 min on an orbital shaker. At last, 100 μL of the sample was transferred to a 96‐well microplate and measured at 560 nm. For the invasion assay, the upper surface of the insert membrane was coated with a uniform layer of dried basement membrane matrix solution. This basement membrane layer serves as a barrier to discriminate invasive cells from non‐invasive cells.

### 2.6 RNA‐sequencing and data analysis

Total RNA was extracted using the TRIzol reagent, and RNA‐sequencing was performed on an Illumina HiSeq 4000 platform. Differentially expressed genes were defined as log2|fold change| ≥ 1 with adjust *p* value less than 0.05. The gene expression patterns in different pathways were analysed using the KEGG pathway database.

### 2.7 Analysis of oxidative stress and lipid peroxidation

Intracellular ROS was measured using a DCFH‐DA probe as previously described.^24^ Briefly, cells were incubated with DCFH‐DA (10 μmol/L) for 30 min at 37°C in the dark, and the fluorescence intensity was measured at an excitation wavelength of 488 nm and an emission wavelength of 525 nm. To measure lipid peroxidation, intracellular malondialdehyde (MDA) was measured using a Lipid Peroxidation Assay Kit (Abcam) according to the manufacturers' instructions. Briefly, supernatants from cell lysates were prepared and incubated with 600 μL of TBA reagent at 95°C for 1 h, and OD values were measured at 532 nm.

### 2.8 Measurements of 12/15‐HETEs in cell medium

Levels of 12/15‐HETEs in cell medium were measured using the 12/15(S)‐HETE ELISA Kit (Abcam) according to the manufacturers' instructions.

### 2.9 Ferrous iron detection

Ferrous iron level was detected using the Iron Assay Kit (Abcam) according to the manufacturers' instructions. Briefly, supernatants from cell lysates were prepared and incubated with 5 μL of Assay Buffer at 37°C for 30 min, and then incubated with 100 μL Iron Probe at 37°C for 1 h in the dark. OD values were measured at 593 nm.

### 2.10 Measurements of GSH and glutathione peroxidase 4 (GPX4) activity

Intracellular GSH level was measured using the GSH Assay Kit (Abcam) according to the manufacturers' instructions. Briefly, supernatants from cell lysates were prepared and incubated with 80 μL Reaction Mix containing 10 μL Substrate Mix A, 10 μL Diluted Enzyme Mix A, 1 μL Enzyme Mix B, 2 μL Enzyme Mix B, 2 μL Substrate Mix B and 55 μL GSH Assay Buffer at room temperature for 1 h and OD values were measured at 450 nm. To measure GPX4 activity, cells were lysed and incubated 7α cholesterol hydroperoxide (100 μmol/L) at 37°C. Then, peroxides were analysed with high‐performance thin‐layer chromatography as previously described.^25^

### 2.11 Quantitative real‐time PCR

Total RNA was extracted using the TRIzol reagent, and reverse transcription as well as quantitative real‐time PCR were performed as previously described.^26^, ^27^, ^28^ Relative mRNA expression was analysed by the 2^‐△△CT^ formula. The primers were as following: ACSL4 forward 5′‐TCTGCTTCTGCTGCCCAATT‐3′ and reverse 5′‐CGCCTTCTTGCCAGTCTTTT‐3′; β‐actin forward 5′‐GGGAAATCGTGCGTGACATT‐3′ and reverse 5′‐GGAACCGCTCATTGCCAAT‐3′.

### 2.12 Database analysis

FLRT2 expression, and its correlation with patients survival and ACSL4 expression in BLCA human bladder cancer database were analysed by the Gene Expression Profiling Interactive Analysis 2 (GEPIA2) database (http://gepia2.cancer‐pku.cn/#index) according to the manufacturers' instructions.

### 2.13 Human samples

Human bladder cancer tissues (Tumour, *n* = 6) and matched adjacent noncancerous tissues (Normal, *n* = 6) were obtained from Renmin Hospital of Wuhan University. The study was approved by the Ethics Committee of Renmin Hospital of Wuhan University (approval no. WDRY2019‐K035), and a written informed consent was obtained from each patient. The specimens were frozen and stored at −80°C until used for western blot analysis.

### 2.14 Statistical analysis

Statistical analyses were performed with SPSS software (version 22.0) and shown as the means ± SD. Comparisons between 2 groups were performed with the two‐tailed Student's *t*‐test, while one‐way analysis of variance (anova) was conducted to compare differences among multiple groups. Survival curves were presented using Kaplan–Meier analysis and the log‐rank test, and Spearman correlation analysis was used for the analysis between FLRT2 and ACSL4. *p* < 0.05 was considered statistically significant.

## 3 RESULTS

### 3.1 FLRT2 expression is reduced in human bladder cancer and negatively correlates with patients survival

**FIGURE 1:**
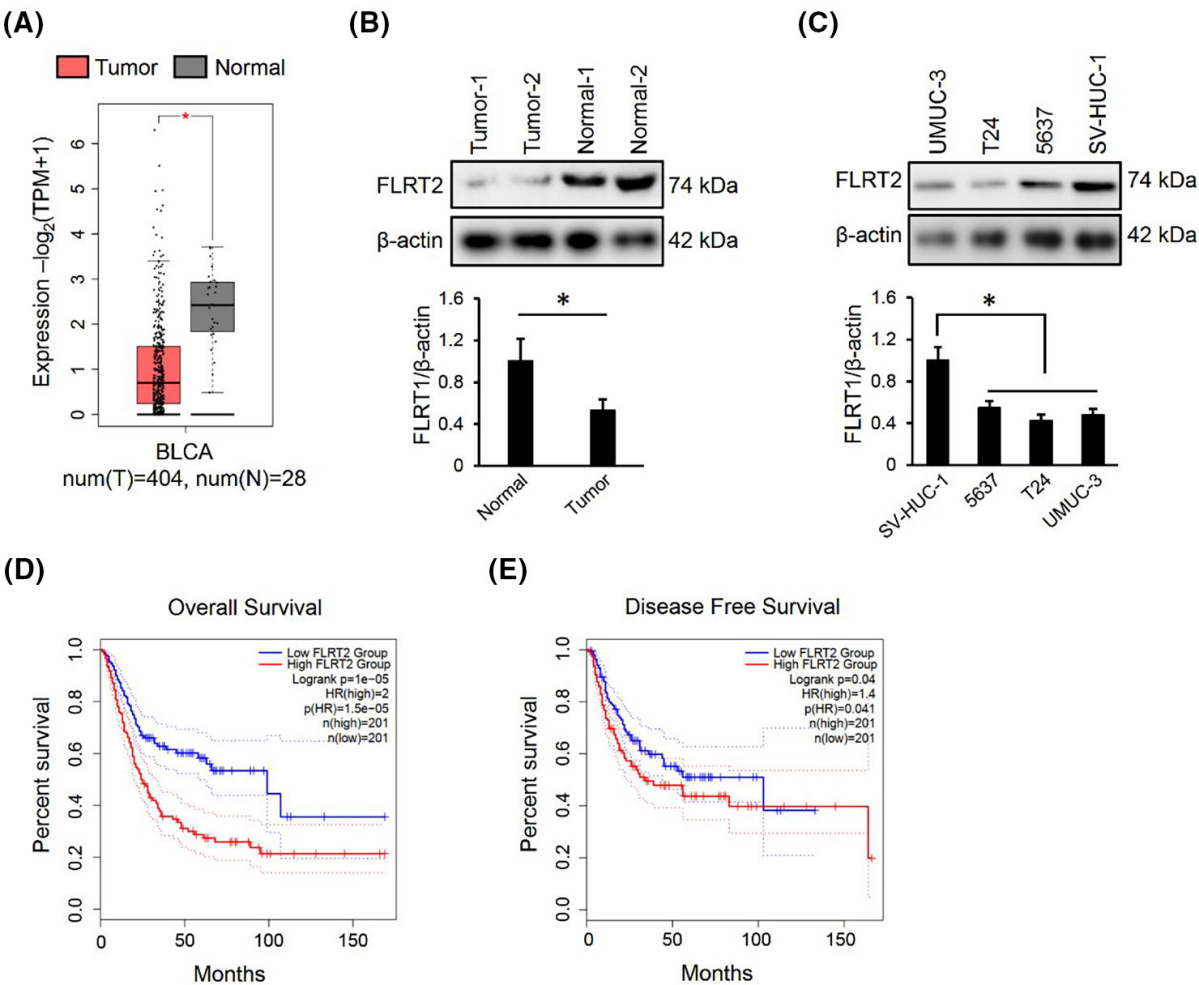
FLRT2 expression is reduced in bladder cancer, and negatively correlates with patients survival. (A) FLRT2 expression in human bladder cancer from BLCA public database (*n* = 404 or 28). (B, C). FLRT2 protein levels in human bladder cancer tissues or cell lines (*n* = 6). (D, E) Kaplan–Meier analysis of the relationship between FLRT2 expression and overall survival rate or disease free survival rate (*n* = 201). All data are presented as the mean ± SD, *p* < 0.05 was considered statistically significant.

To explore the function of FLRT2, we first compared its expression in tumour with matched normal tissues using the BLCA public database. As displayed in Figure 1A, FLRT2 level was decreased in human bladder cancer. Consistently, we also found that human bladder cancer tissues displayed lower protein levels of FLRT2 (Figure 1B). Besides, protein quantification assay also detected a decreased FLRT2 protein in bladder cancer cells compared with the normal SV‐HUC‐1 cell (Figure 1C). From the BLCA public database, we also found that bladder cancer patients possessing higher FLRT2 level exhibited lower overall survival together with disease free survival rates (Figure 1D,E). Collectively, our findings reveal that FLRT2 expression is reduced in human bladder cancer and negatively correlates with patients survival.

### 3.2 FLRT2 overexpression inhibits the malignant phenotypes of human bladder cancer cells

**FIGURE 2:**
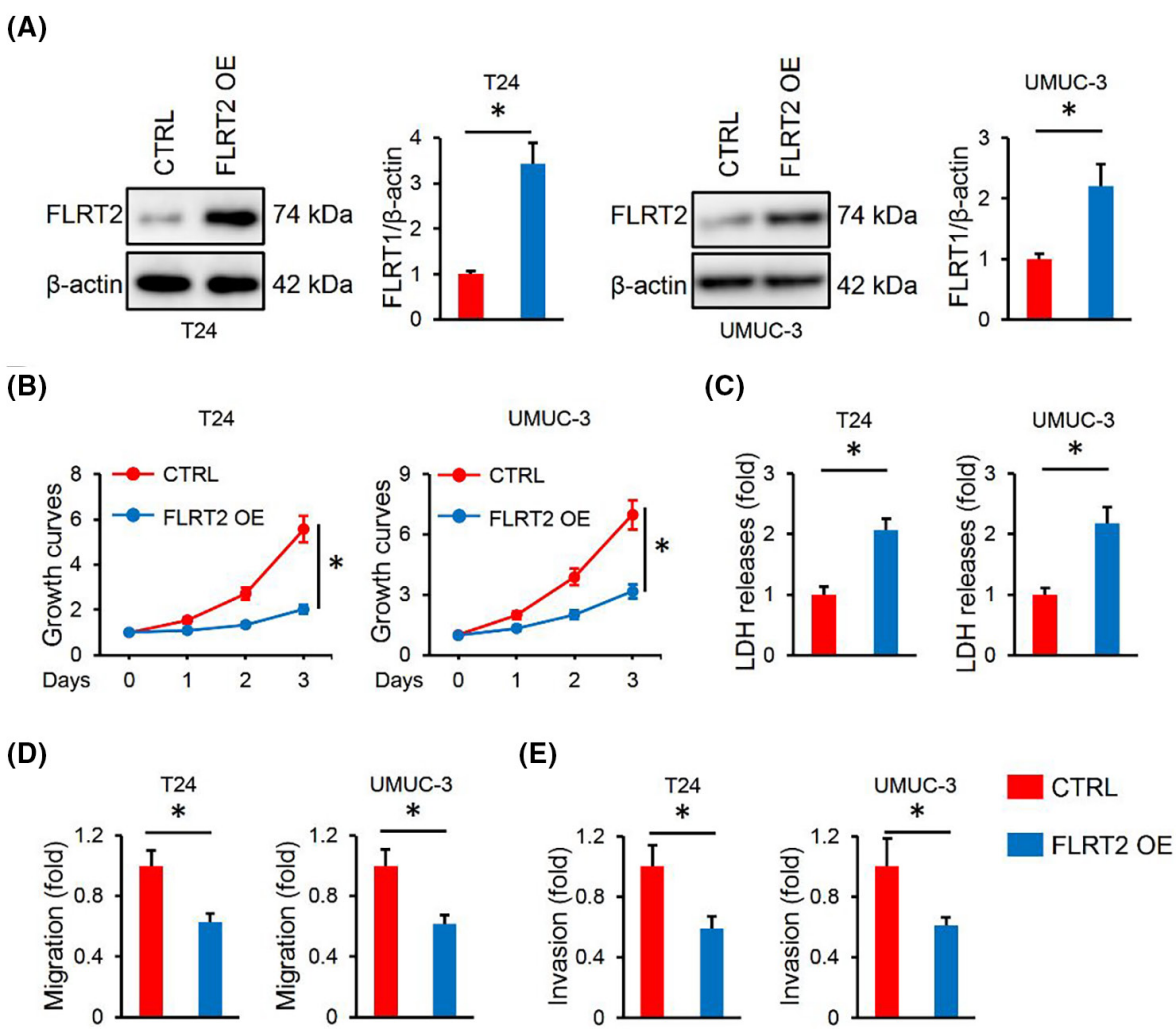
FLRT2 overexpression inhibits the malignant phenotypes of human bladder cancer cells. (A) FLRT2 protein levels in T24 or UMUC‐3 cells with or without FLRT2 overexpression (*n* = 6). (B) CCK‐8 assay was performed to detect cell survival in FLRT2‐overexpressed or CTRL human bladder cancer cells (*n* = 6). (C) LDH releases were measured to evaluate cell death (*n* = 6). (D, E) Cell migration and invasion in FLRT2‐overexpressed or CTRL human bladder cancer cells (*n* = 6). All data are presented as the mean ± SD, *p* < 0.05 was considered statistically significant.

Next, we overexpressed FLRT2 in T24 and UMUC‐3 cells, and the efficiency was provided in Figure 2A. CCK‐8 assay showed decreased proliferation in FLRT2‐overexpressed cells in comparison with the non‐overexpressed groups (Figure 2B). In contrary, the degree of death was induced after overexpressing FLRT2, as indicated by the increased LDH releases (Figure 2C). Meanwhile, we found that FLRT2 overexpression also significantly inhibited the migrative and invasive capacities of the two cancer cells (Figure 2D,E). Collectively, we demonstrate that FLRT2 overexpression inhibits the malignant phenotypes of human bladder cancer cells.

### 3.3 FLRT2 silence facilitates the malignant phenotypes of human bladder cancer cells

**FIGURE 3:**
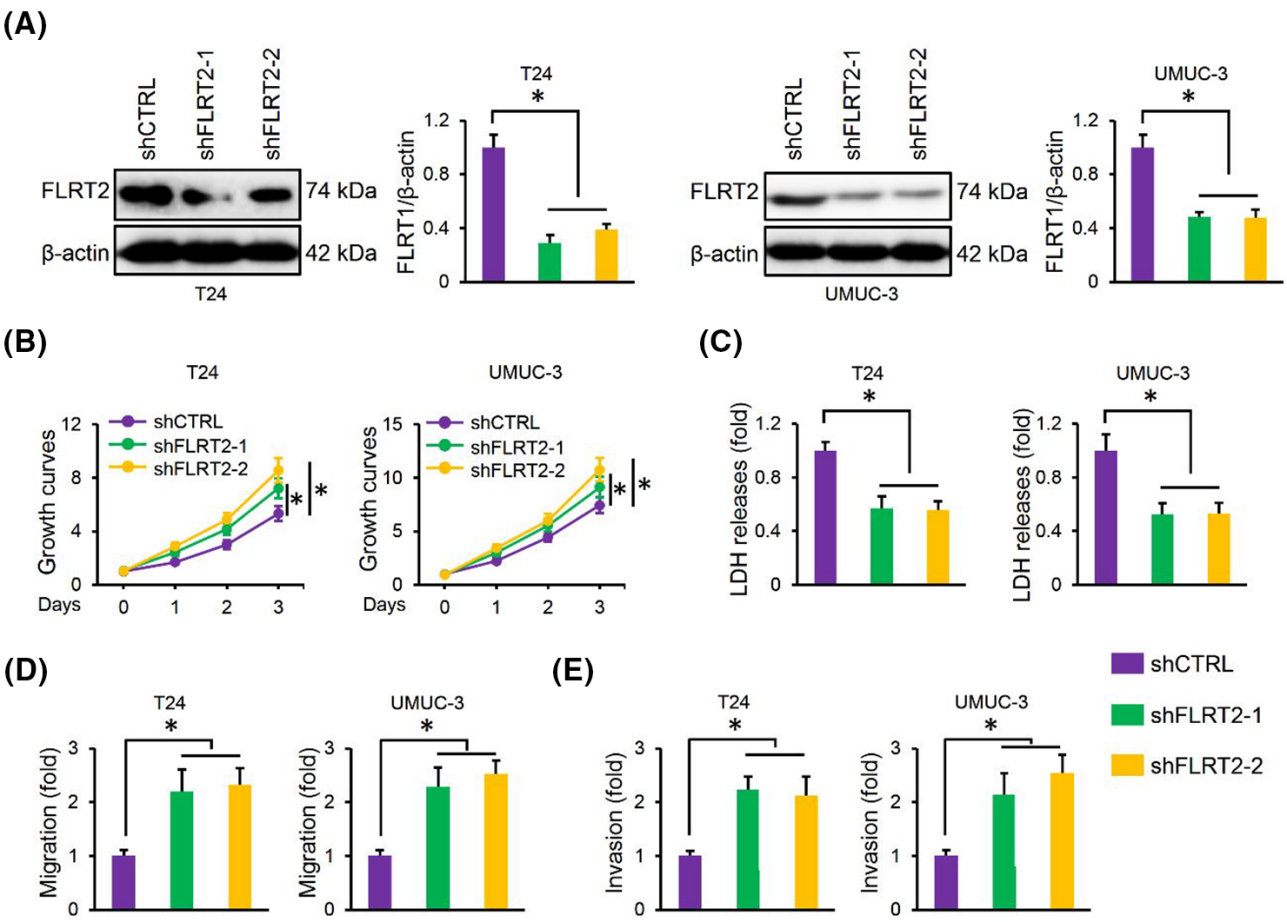
FLRT2 silence facilitates the malignant phenotypes of human bladder cancer cells. (A) FLRT2 protein levels in T24 or UMUC‐3 cells with or without FLRT2 silence (*n* = 6). (B) CCK‐8 assay was performed to detect cell survival in FLRT2‐silenced or CTRL human bladder cancer cells (*n* = 6). (C) LDH releases were measured to evaluate cell death (*n* = 6). (D, E) Cell migration and invasion in FLRT2‐silenced or CTRL human bladder cancer cells (*n* = 6). All data are presented as the mean ± SD, *p* < 0.05 was considered statistically significant.

Furthermore, we silenced FLRT2 in human bladder cancer cells, and the protein levels of FLRT2 were presented in Figure 3A. As expected, FLRT2 silence remarkably increased the proliferation of T24 and UMUC‐3 cells, while the death was inhibited (Figure 3B,C). Meanwhile, we found that FLRT2 silence also enhanced the migrative and invasive capacities of the two cells (Figure 3D,E). Collectively, we demonstrate that FLRT2 silence facilitates the malignant phenotypes of human bladder cancer cells.

### 3.4 FLRT2 overexpression promotes ferroptosis of human bladder cancer cells

**FIGURE 4:**
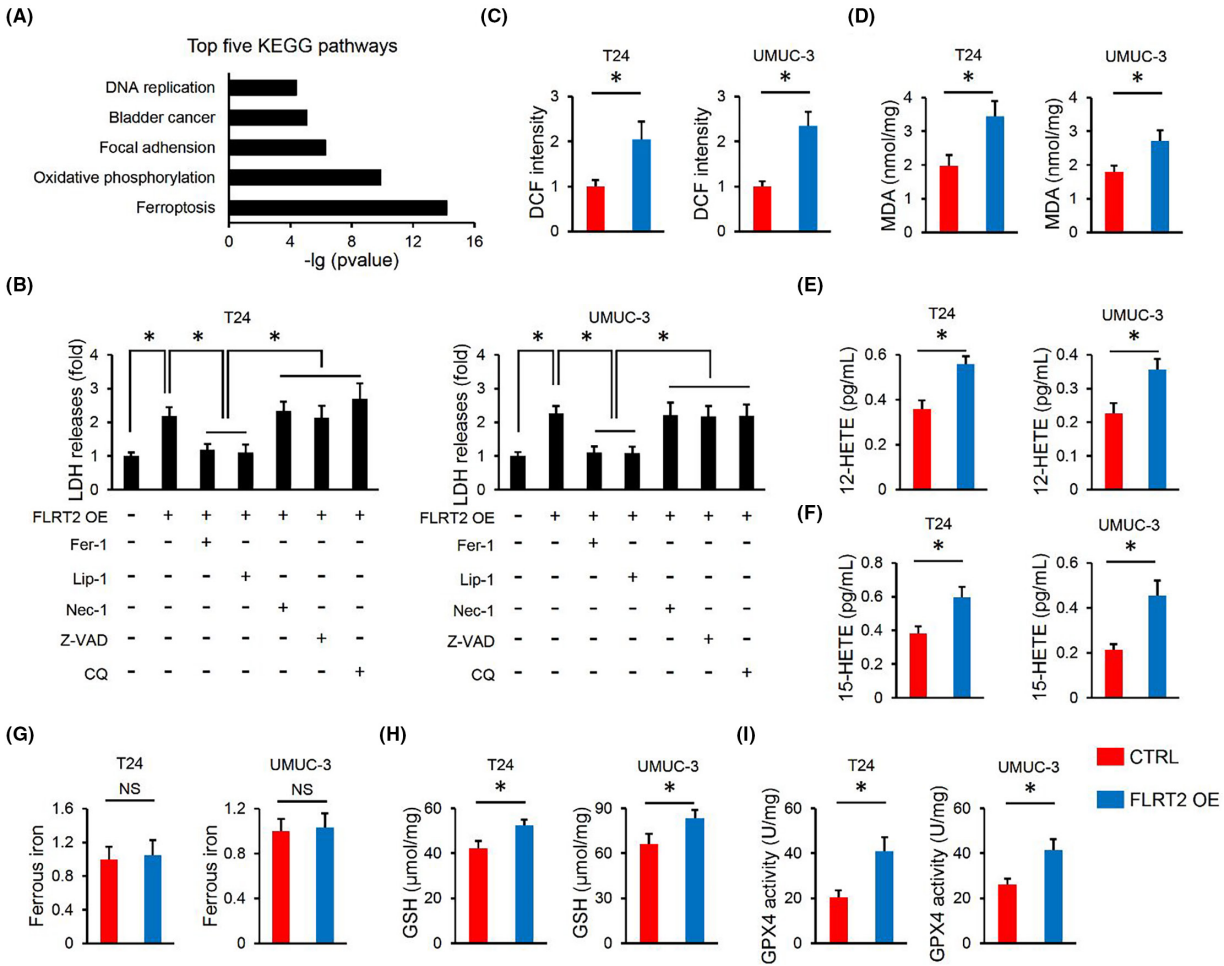
FLRT2 overexpression promotes ferroptosis of human bladder cancer cells. (A) KEGG analysis of the top five pathways altered in FLRT2‐silenced T24 cells (*n* = 3 or 6). (B) LDH releases were measured to evaluate cell death (*n* = 6). (C, D) ROS generation and lipid peroxidation in FLRT2‐overexpressed or CTRL human bladder cancer cells (*n* = 6). (E, F) Levels of 12/15‐HETEs in cell medium (*n* = 6). (G) Levels of ferrous iron (*n* = 6). (H, I) Levels of GSH and GPX4 activity in FLRT2‐overexpressed or CTRL human bladder cancer cells (*n* = 6). All data are presented as the mean ± SD, *p* < 0.05 was considered statistically significant. NS indicates no significance.

Then, RNA‐sequencing was performed to clarify the underlying mechanism by which FLRT2 regulated the tumorigenesis of human bladder cancer. KEGG analysis revealed that ferroptosis in T24 cells was significantly regulated by FLRT2 silence (Figure 4A). To further clarify the involvement of ferroptosis, FLRT2‐overexpressed cells were treated with the inhibitors of ferroptotic, necroptotic, apoptotic or autophagic cell deaths. Interestingly, only the ferroptosis inhibitors effectively inhibited the induction of cell death by FLRT2 overexpression (Figure 4B). Ferroptosis is an oxidative, iron‐dependent form of cell death.^29^ As expected, FLRT2 overexpression significantly increased the levels of ROS and MDA in human bladder cancer cells (Figure 4C,D). During ferroptosis, polyunsaturated fatty acids in cell membranes are oxidized into various metabolites, including HETEs.^30^ Herein, we found that FLRT2 overexpression increased the levels of 12/15‐HETEs in the medium of T24 and UMUC‐3 cells (Figure 4E,F). Iron overload contributes to ferroptosis; yet, our findings showed that FLRT2 did not affect intracellular iron level in human bladder cancer cells (Figure 4G). Inactivation of GSH/GPX4‐dependent antioxidant defences contributes to the accumulation of toxic lipid ROS, thereby inducing ferroptosis.^30^ Unexpectedly, GSH level and GPX4 activity were slightly but significantly elevated in FLRT2‐overexpressed bladder cancer cells, suggesting a compensatory protection of GSH/GPX4 (Figure 4H,I). Collectively, we demonstrate that FLRT2 overexpression promotes ferroptosis of human bladder cancer cells.

### 3.5 FLRT2 silences suppresses ferroptosis of human bladder cancer cells

**FIGURE 5:**
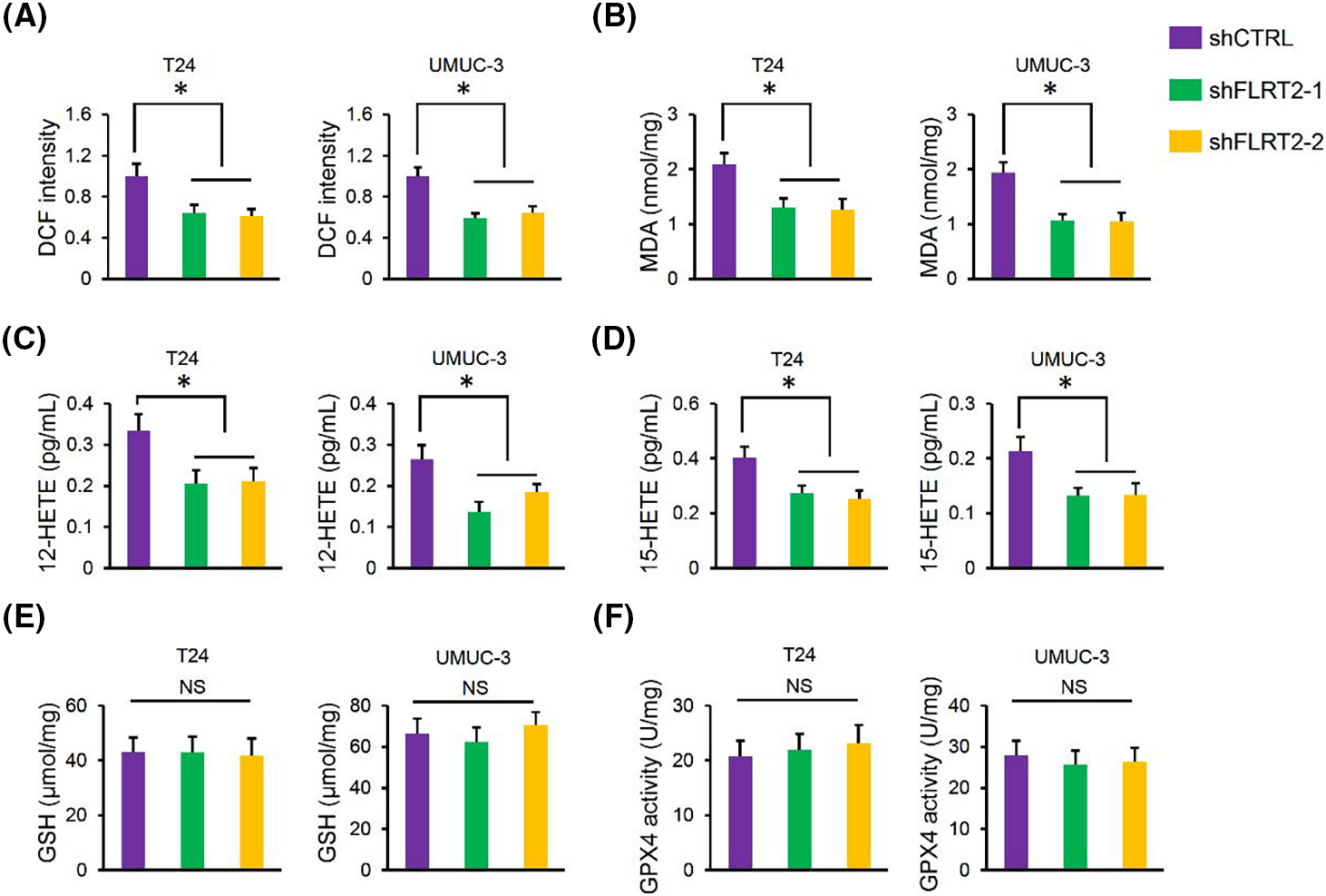
FLRT2 silences suppresses ferroptosis of human bladder cancer cells. (A, B) ROS generation and lipid peroxidation in FLRT2‐silenced or CTRL human bladder cancer cells (*n* = 6). (C, D) Levels of 12/15‐HETEs in cell medium (*n* = 6). (E, F) Levels of GSH and GPX4 activity in FLRT2‐silenced or CTRL human bladder cancer cells (*n* = 6). All data are presented as the mean ± SD, *p* < 0.05 was considered statistically significant. NS indicates no significance.

In contrast, FLRT2 silence significantly decreased the levels of ROS and MDA in human bladder cancer cells (Figure 5A,B). Meanwhile, the levels of 12/15‐HETEs in the medium were also decreased by FLRT2 silence (Figure 5C,D). Yet, GSH level and GPX4 activity were unaffected by FLRT2 silence in human bladder cancer cells (Figure 5E,F). Collectively, we demonstrate that FLRT2 silences suppresses ferroptosis of human bladder cancer cells.

### 3.6 FLRT2 triggers ferroptosis through elevating ACSL4

**FIGURE 6:**
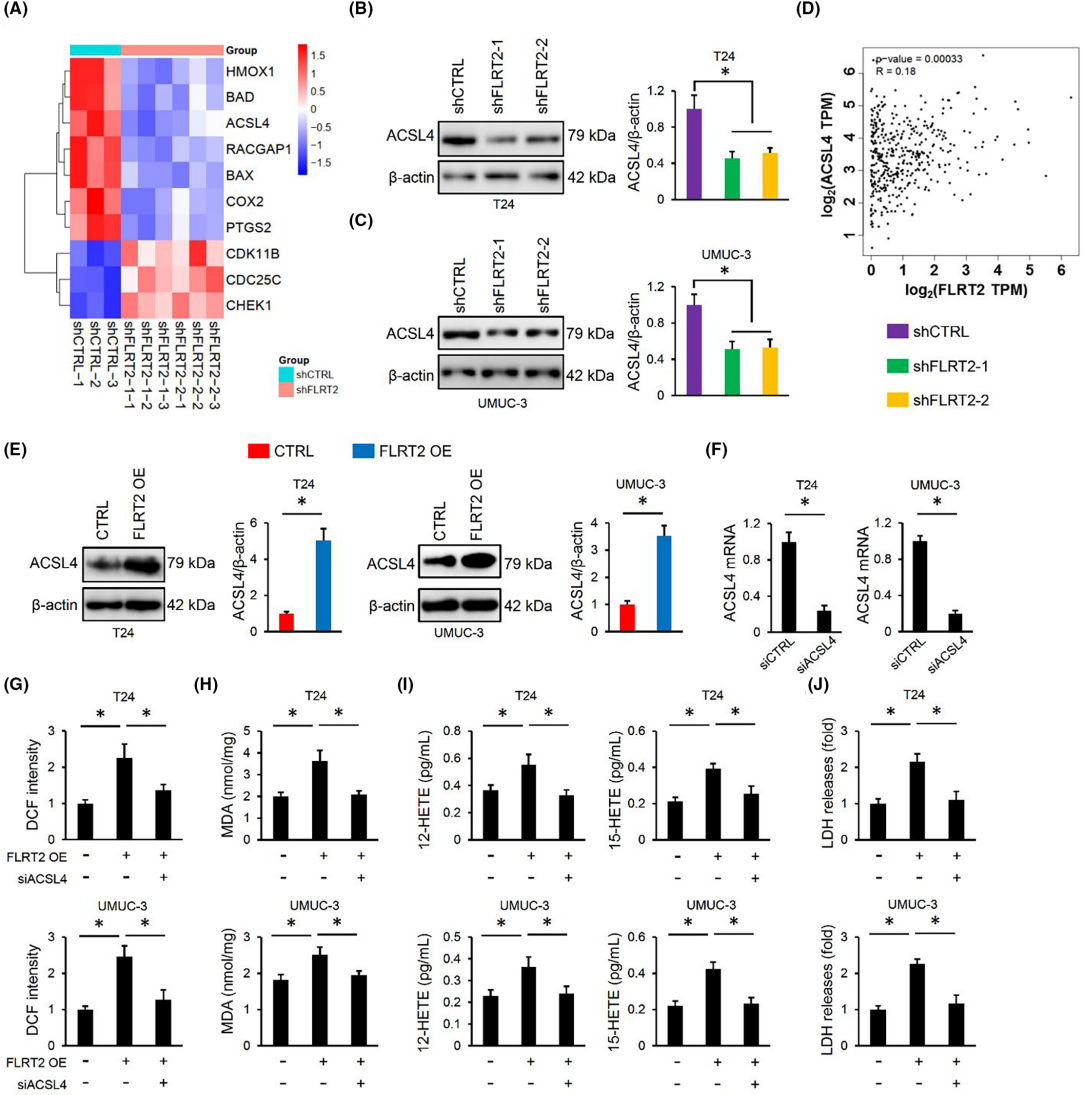
FLRT2 triggers ferroptosis through elevating ACSL4. (A) Heatmaps of the differentially expressed genes (*n* = 3). (B, C) ACSL4 protein levels in T24 or UMUC‐3 cells with or without FLRT2 silence (*n* = 6). (D) Spearman correlation analysis was used for the analysis between FLRT2 and ACSL4. (E) ACSL4 protein levels in T24 or UMUC‐3 cells with or without FLRT2 overexpression (*n* = 6). (F) ACSL4 mRNA level in T24 or UMUC‐3 cells with or without ACSL4 silence (*n* = 6). (G, H) ROS generation and lipid peroxidation in FLRT2‐overexpressed or CTRL human bladder cancer cells with or without ACSL4 silence (*n* = 6). (I) Levels of 12/15‐HETEs in cell medium (*n* = 6). (J) LDH releases were measured to evaluate cell death (*n* = 6). All data are presented as the mean ± SD, *P* < 0.05 was considered statistically significant.

The above findings revealed that FLRT2 might regulate ferroptosis of human bladder cancer cells through regulating lipid peroxidation, instead of regulating iron metabolism or GSH/GPX4‐dependent antioxidant defences. By analysing the RNA‐sequencing data, we found that ACSL4, an enzyme required for arachidonic acid metabolism, lipid peroxidation and ferroptosis, was significantly decreased in FLRT2‐silence T24 cells (Figure 6A).^31^, ^32^ As expected, ACSL4 protein levels were also decreased in FLRT2‐silenced cells (Figure 6B,C). Meanwhile, FLRT2 expression was also positively associated with ACSL4 level in the BLCA public database (Figure 6D). In addition, FLRT2 overexpression remarkably increased ACSL4 protein levels in the two cells (Figure 6E). To clarify the importance of ACSL4, we silenced ACSL4 in T24 and UMUC‐3 cells (Figure 6F). As expected, ACSL4 silence significantly blocked the generations of ROS and MDA in FLRT2‐overexpressed T24 and UMUC‐3 cells (Figure 6G,H). Accordingly, the increased levels of 12/15‐HETEs in the medium of FLRT2‐overexpressed cells were also decreased by ACSL4 silence (Figure 6I). Moreover, FLRT2 overexpression‐induced cell death was also inhibited by ACSL4 silence (Figure 6J). Collectively, we demonstrate that FLRT2 triggers ferroptosis through elevating ACSL4.

## 4 DISCUSSION

Bladder cancer is emerging as a common human cancer, and the prognosis is poor. In this study, FLRT2 expression was found to be reduced in human bladder cancer, and higher FLRT2 level predicted lower survival rate. FLRT2 overexpression inhibited, while FLRT2 silence facilitated tumour cell growth, migration and invasion. Mechanistic studies revealed that FLRT2 elevated ACSL4 expression, increased lipid peroxidation and subsequently facilitated ferroptosis of human bladder cancer cells. Overall, we demonstrate that FLRT2 suppresses bladder cancer progression through inducing ACSL4‐mediated ferroptosis.

Cell death is essential for tissue balance, including human tumours. Ferroptosis, triggered by iron‐dependent accumulation of toxic lipid ROS, is a newly identified form of programmed cell death that is distinct from apoptosis, necrosis, autophagy and other forms of cell death.^29^ Ferroptosis is implicated in the tumorigenesis of multiple tumours, such as hepatocellular carcinoma, lung cancer and glioma. Tang et al.^17^ previously showed that inducing ferroptosis significantly suppressed the malignant phenotype of non‐small cell lung cancer cells in vivo and in vitro. Recent studies have also identified an indispensable role of ferroptosis in the progression of bladder cancer.^7^, ^8^ We herein demonstrated that FLRT2 overexpression facilitated ferroptosis of bladder cancer cells, thereby inhibiting tumour cell proliferation, migration and invasion. ACSL4 is involved in lipid peroxidation, and facilitate the esterification of arachidonoyl and adrenoyl into phosphatidylethanolamine that are closely related to ferroptosis.^32^ Li et al.^31^ previously determined that ACSL4 activation enhanced ferroptosis, thereby aggravating intestinal ischemia/reperfusion injury. In contrast, the inhibition of ACSL4 effectively prevented lipid peroxidation and ferroptosis.^33^, ^34^ We herein defined ACSL4 as a target of FLRT2. FLRT2 overexpression elevated, while FLRT2 silence reduced ACSL4 protein expression in human bladder cancer cells. Knocking down ACSL4 abrogated lipid peroxidation and ferroptosis in FLRT2‐overexpressed T24 and UMUC‐3 cells. Yet, the specific mechanism by which FLRT2 regulated ACSL4 remains unclear. FLRT2 was identified as a transmembrane protein, and interacted with UNC5, fibronectin and FGFR2 to regulate various biological process.^9^, ^35^, ^36^ Whether FLRT2 regulated ACSL4 through direct interaction or other partners should be dissected in future studies.

We demonstrate that FLRT2 elevates ACSL4 expression and subsequently facilitates lipid peroxidation and ferroptosis, thereby inhibiting the malignant phenotype of human bladder cancer cells. Overall, we identify FLRT2 as a tumour suppressor gene.

## AUTHOR CONTRIBUTIONS

**Pengcheng Jiang:** Conceptualization (equal); data curation (equal); methodology (equal); software (equal); validation (equal); writing – original draft (equal). **Jin‐Zhuo Ning:** Conceptualization (equal); data curation (equal); methodology (equal); visualization (equal). **Weimin Yu:** Methodology (equal); software (equal). **Ting Rao:** Data curation (equal); investigation (equal); methodology (equal); software (equal). **Yuan Ruan:** Conceptualization (equal); investigation (equal); supervision (equal); visualization (equal). **Fan Cheng:** Funding acquisition (equal); project administration (equal); supervision (equal).

## FUNDING INFORMATION

This work was funded by the National Natural Science Foundation of China [No. 82170775; No. 82100806].

## CONFLICT OF INTEREST STATEMENT

The authors declare no conflicts of interest.

## Data Availability

All data that support the findings in this study are available from the corresponding author upon reasonable request.
